# Mitigating the Impact of the Novel Coronavirus Pandemic on Neuroscience and Music Research Protocols in Clinical Populations

**DOI:** 10.3389/fpsyg.2020.02160

**Published:** 2020-08-28

**Authors:** Efthymios Papatzikis, Fathima Zeba, Teppo Särkämö, Rafael Ramirez, Jennifer Grau-Sánchez, Mari Tervaniemi, Joanne Loewy

**Affiliations:** ^1^Department of Psychology, Canadian University Dubai, Dubai, United Arab Emirates; ^2^Latifa Women and Children Hospital, Dubai, United Arab Emirates; ^3^Cognitive Brain Research Unit, Department of Psychology and Logopedics, Faculty of Medicine, University of Helsinki, Helsinki, Finland; ^4^Music and Machine Learning Lab, Universitat Pompeu Fabra, Barcelona, Spain; ^5^Cognition and Brain Plasticity Unit, Bellvitge Biomedical Research Institute, L’Hospitalet de Llobregat, Spain; ^6^Escola Universitària d’Infermeria i Teràpia Ocupacional de Terrassa, Autonomous University of Barcelona, Terrassa, Spain; ^7^Cicero Learning Network, Faculty of Educational Sciences, University of Helsinki, Helsinki, Finland; ^8^The Louis Armstrong Center for Music and Medicine, Mount Sinai Beth Israel, Icahn School of Medicine, New York, NY, United States

**Keywords:** COVID-19, music and neuroscience, music therapy, music and neuroscience research protocols, research crisis response

## Abstract

The COVID-19 disease and the systemic responses to it has impacted lives, routines and procedures at an unprecedented level. While medical care and emergency response present immediate needs, the implications of this pandemic will likely be far-reaching. Most practices that the clinical research within neuroscience and music field rely on, take place in hospitals or closely connected clinical settings which have been hit hard by the contamination. So too have its preventive and treatment measures. This means that clinical research protocols may have been altered, postponed or put in complete jeopardy. In this context, we would like to present and discuss the problems arising under the current crisis. We do so by critically approaching an online discussion facilitated by an expert panel in the field of music and neuroscience. This effort is hoped to provide an efficient basis to orient ourselves as we begin to map the needs and elements in this field of research as we further propose ideas and solutions on how to overcome, or at least ease the problems and questions we encounter or will encounter, with foresight. Among others, we hope to answer questions on technical or social problems that can be expected, possible solutions and preparatory steps to take in order to improve or ease research implementation, ethical implications and funding considerations. Finally, we further hope to facilitate the process of creating new protocols in order to minimize the impact of this crisis on essential research which may have the potential to relieve health systems.

## Introduction

The novel coronavirus disease (COVID-19) and the consequent systemic responses have impacted lives, systems and procedures at an unprecedented level. While medical care and emergency responses are the need of the hour, the implications of this pandemic are more far-reaching having direct consequences on health sciences and research. Neuroscience and music research, and specially studies of music therapy, often take place in hospitals and health services. However, daily practices at these institutions have been reorganized to provide treatments for patients diagnosed with COVID-19, as well as to prevent the spread of the disease among professionals and patients with other conditions. As a result, relevant to music and neuroscience clinical research protocols have been subsequently temporarily suspended, put in jeopardy, or have been altered.

In this context, we would like to present and then address the problems – related to methodological, technical, and potential psychosocial ramifications – that music and neuroscience researchers in clinical settings may be facing. We do so by consolidating the insights gleaned from an international online forum attended by clinicians and researchers in the field of music and neuroscience. It is hoped that this effort will provide ideas, answers to questions, and possible solutions to problems in the ongoing or anticipated impact of COVID-19 on active research protocols, thereby improving research implementation, ethical implications and subsequent funding considerations. We further hope to facilitate the process of creating new protocols in order to minimize the impact of this crisis on essential research which may have the potential to relieve health systems.

## The Discussion Context

The pandemic crisis has brought a myriad of new challenges to research practices in the international research community. It has also seemingly fostered the potential for a pioneering of new insights, beckoning professionals to examine how they can interact and solve problems in their respective scientific fields in novel ways. Many such insights were addressed during an online interaction held on April 15th, 2020. The forum, employing the Webex platform, provided the opportunity for a discussion of these issues and potential solutions related to active music and neuroscience clinical research protocols during the COVID-19 crisis period. Live polls were also conducted through the Webex platform, where participants could choose to answer questions posed by the organizers through an interactive platform. The discussion panel was comprised of five clinicians and researchers in this specific field joining from Finland, the United States, Spain and the United Arab Emirates. The discussion was attended by 392 participants from 41 different countries (for a breakdown on participants’ demographics please see Table 1). There was a mix of academics, researchers and students of all levels. Among those who responded to the polls (60.15%) 35% were actively involved in music and neuroscience research. Similarly, the polls reported that 29% were planning to research in the area, and another 43% were contemplating researching in the areas of neuroscience and music. Each of the panelists presented their viewpoints, while sharing their own distinct crisis reality each through a unique lens. The themes that emerged were explored, categorized and expanded on by the authors who were also the panelists. Six major themes were revealed following an informal analysis of the discourse after the end of the teleconference. These were based on the panel discussion, and the questions posed from the participants, as well as the live survey-polls.

**TABLE 1:** Breakdown of participants’ demographics.

Argentina	1	Latvia	1
Brazil	1	Malaysia	1
Canada	16	Mexico	2
China	1	Myammar	1
Colombia	2	Netherlands	3
Cyprus	2	Nigeria	1
Denmark	2	Poland	1
Ecuador	1	Portugal	4
Egypt	1	Qatar	1
Estonia	3	Russia	1
Finland	4	Singapore	1
France	5	South Africa	4
Georgia	1	Spain	13
Germany	4	Sri Lanka	1
Greece	19	Switzerland	3
Hong-Kong	2	Taiwan	1
Hungary	2	Turkey	2
India	17	United Arab Emirates	87
Ireland	1	United Kingdom	16
Italy	8	United States	76
Jordan	1	Unknown	78

## The Discussion Themes

The six themes presented below, roughly outline the COVID-19 crisis research situation, while also bringing to the forefront some crucial points to consider for future planning.

### Factors Directly Affecting Protocols

#### Changed Timelines

Governmental policies facing the COVID-19 pandemic led to the cessation of many professional activities in the majority of countries, either because “essential activities” were the only ones permitted to take place, or because restrictions interfered with the usual and customary development of these activities. In the field of music, music therapy and neuroscience research, these governmental policies have a direct effect on data collection. Researchers in this field may continue designing studies, analyzing data and/or preparing their manuscripts, however, some on-going and about-to-start studies were forced to stop their protocols as either researchers or participants are unable to get to the lab, hospital or health service clinic. This is a critical issue particularly for clinical trials which follow specific, pre-set protocols. Researchers are faced with difficult choices and fast decisions on questions such as “can the music intervention be altered so that it can be implemented through remote communication” and “how does stopping and restarting the intervention later affect the design of the study.”

#### Technological Challenges in Adapting to Changes

Music-based interventions are complex as they have several interacting components (Craig et al., 2008). Adaptation of intervention protocols require a theoretical understanding of how these components contribute to the treatment success; which components are essential; and how possible modifications to remotely implement the intervention would affect the participants and the study outcomes. Further collaborations and interaction with other fields such as engineering/information technology are needed to effectively adapt interventions for their virtual or remote implementation. Apart from the technical challenges, new ways of delivery also posit several ethical challenges, such as ensuring the safe and secure collection, and storage of clinical data, as well as data potentially gathered at participants’ homes (Stanberry, 2006; Kaplan and Litewka, 2008). Moreover, implementation of adapted interventions requires piloting and testing procedure feasibility, processes that take time and can delay a rapid response to this situation. For on-going studies, adapting an intervention protocol would force researchers to analyze the data of those participants who received the intervention in an adapted form, separately from those who participated under normal clinical conditions.

#### Loss of Control Over Confounding Variables

Prolonged breaks in interventions and treatments if the study is interrupted and restarted later may have direct consequences on research quality. One of the main challenges when restarting a protocol will be to ensure that the conditions related to the setting, professionals and procedures remain as similar as possible to the conditions set before the crisis. However, it is uncertain how hospitals and health services are going to operate in the future. Researchers will have to analyse these contexts and control their changes to avoid potential confounding variables.

Confounds can also occur due to the nature of the populations that are usually involved in neuroscience and music studies. Research in music, music therapy and neuroscience often involve vulnerable populations. These populations are inevitably prone to being affected by isolation, and the lack of activity and rehabilitative treatments during this period, as well as by the predicted future economic crisis. There may be long-term negative physical, cognitive and psychosocial consequences in these vulnerable populations, which will force researchers to redefine music-based interventions and protocols in the future. Researchers may be asked to address new needs that may arise, consequently changing how protocols for treatment are instituted and measured.

### Factors Directly Affecting the Sample

While the domain of neuroscience and music promotes exploratory studies on brain development (for example Papatzikis, 2017; Putkinen et al., 2019; for a review see Tervaniemi et al., 2018), quite a lot of research in the field is rehabilitative in nature (Särkämö et al., 2016; Cheever et al., 2018). The rehabilitative studies in the field include recovery from stroke and brain injury as well as other neurological and neurodegenerative disorders. Additionally, the specific field deals with neurodiverse research population paradigms focusing on autism, ADHD and pre-term infants (Loewy et al., 2013).

One apparent element of the discussion was that populations with high vulnerability to the disease cannot physically participate in active research at the moment, due to possible coronavirus exposure, while having even more difficulty safely socializing or traveling to attend research sessions if needed. It was also noted that especially research focusing on music group interventions can no longer be conducted in many places around the world due to the restrictions on mobility and allowed size of any type of group gathering. Tragically, in ongoing clinical trials and longitudinal studies, it can also be anticipated that there will be participants who dropout of studies because of COVID-19, either directly because of illness or death or indirectly because of changes in life situation. While further recruitment might be feasible later on, at the very epicenter of the pandemic, it is quite difficult to proceed with it at present. If the pandemic and the state of emergency continue for long, it may also negatively affect the general willingness and motivation of people, especially the high risk groups most affected, to participate in research as subjects, as it may be seen as unsafe or abortive.

In parallel, it was noted that the sample recruitment difficulty will most definitely affect the context of music and neuroeducation, too – a closely related field with challenges similar to those met in music rehabilitation. Its importance is intrinsic in those studies which aim at revealing brain bases for the development of music skills, and which also explore the existence of transfer skills influenced by music activities. These studies recruit participants from newborns until adolescence mostly in longitudinal paradigms, and they face similar constrains and uncertainties in the current situation.

### Factors Due to the Setting

Music, music therapy and neuroscience research most frequently occurs in clinical settings. With some hospitals placing restrictions on “non-essential” services, research for many has come to a grinding halt. For example, neonatal intensive care units (NICUs) are a common setting in which to conduct music, music therapy and neuroscience research on infants. In adult neurological and neurodegenerative disorders, such as stroke and dementia, much research takes place in the context of rehabilitation units and care homes. In the COVID-19 pandemic, these facilities are now among the most isolated units as their clinical populations are highly vulnerable and at high risk for contagion, and are therefore out of bounds when it comes to participating in music intervention research. The cruel irony is that especially in this situation, music could actually be a most valuable tool for many to cope with the stress, loneliness, and social exclusion caused by the quarantine-like isolation measures imposed by COVID-19 management. With the ICUs being filled up and overloaded, there is no longer the time or patience to do music research, even though music-based intervention might improve not only patients but also professionals’ well-being.

Regarding the methodological side, music and neuroscience research typically utilizes neuroimaging equipment and facilities, such as electro- or magnetoencephalography (EEG/MEG) and magnetic resonance imaging (MRI). This equipment is often located within the hospital setting. Therefore, physical access to these facilities by researchers is now not only limited by more stringent hospital safety policies but also because in most affected countries available resources are devoted to patients diagnosed with COVID-19. Also, performing the measurements entails being in close physical proximity with the participant (e.g., when attaching EEG electrodes or the MRI head coil) which is not possible due to the need to keep the minimum physical distance, typically specified as 2 meters in the COVID-19 guidelines. While remote or online data collection, for example with e-forms or through various video chat apps, is an option in many fields of research, it is unfortunately not possible in applied neuroscience.

### Interfering Research Variables

Like all experimental research, research in music, music therapy, and neuroscience is carefully structured to ensure the reliability, integrity, and stability of findings. This includes controlling for various intervening factors pertaining to individual variability, stimuli characteristics, music delivery and environmental context (Hunt, 2015) which may affect the results and their interpretation and generalizability. With ongoing research, these factors have been controlled and planned in advance, especially in pre-post design models, where the pre-assessment provides a baseline and any changes (outcomes) are then considered attributable to the effects of the music intervention or activity.

In ongoing clinical trials or other longitudinal studies, the COVID-19 situation, and the mobility and social restrictions posed by it, do not only affect the protocol and sample characteristics (see above) but can also have a broader personal and social impact, which introduces a number of factors that can potentially interfere or bias the longitudinal results of the outcome measures, such as questionnaires on mood and quality of life and cognitive tests measuring attention and memory. For example, the pervasive fear of contracting the COVID-19 virus can increase anxiety. Being socially isolated from friends and family and facing uncertainty about the future can cause stress, rumination, and depression. Restriction from usual participation in social activities and hobbies (including group musical hobbies, such as singing in a choir or playing in a band) induces social deprivation or impoverishes one’s personal environment and lifestyle, which has broad psychological effects on the individual.

Moreover, the economic crisis expected as a consequence of this pandemic will stress the role of social determinants on health, which should be taken into account as interfering variables. In studies comparing intervention and control groups, all of these factors of course affect both groups and thus balance out, but the problem is that they can be so pervasive and strong that they mask out any potential intervention effects. Understanding that the mental state of the population may have changed, baseline assessments may no longer be indicative of current circumstances. Therefore, even if research could go forward, it is at the risk of contamination from confounding variables.

### Resources

With focus on the immediate health and economic outcomes of the pandemic, the shift for hospitalists and most health care providers is now seemingly on managing and coping with the pandemic, rather than on resource-building, *per se*. There may be a lack of empathy from governing bodies for research at this time, because the decision-makers may not see the value of research in the face of more pressing immediate matters. As a result, funding is likely to be – and is already being – redirected toward research dealing directly or indirectly with COVID and its public health outcomes.

Over a longer period, the economic crises that may be imminent could lead to a general reduction on the level of science funding, which, in turn, may affect prioritization of where the reduced funding is allocated. In the ensuing funding competition, smaller, more niched areas of research, including music neuroscience, may take the heaviest tolls. In the meantime, considerations arise for any data collection delays as research timelines are severely affected. This situation would force researchers to negotiate with institutions, decision-makers and funding agencies for new timelines and extended access to protocol resources.

The funders and policy-makers need to be convinced regarding the special relevance for this research during this time, and how instituting and advocating for music as a meaningful and most of all easy, accessible tool to implement in the clinical settings, is necessary. Now more than ever, people need the kind of support provided by music since it can alleviate the impact of isolation and address its cognitive, emotional (Ramirez et al., 2015) and social consequences. Those who may be more vulnerable to isolation such as children with neurodevelopmental disorders and older adults with neurodegenerative disorders are apt to suffer the most, and therefore might benefit the most from such research (Grau-Sánchez et al., 2020).

On another note, some clinical trials, more particularly those involving a longer intervention period, may benefit from resources and treatment adaptation. Maintaining subject treatment strategies may afford subjects and researchers achievable, sustainable status when adaption to a web-based forum can be accomplished. Notably, any intervention that is altered in any way needs to be accounted for and ultimately identified in the data collection process and approved by the IRB (Internal Review Board). For example, the findings of music therapy forums involving groups that sing – addressing the impact of community on outcomes such as aphasia, mood and quality of life – may offer some surprises in the data analyses when outcomes are finally calculated.

One might suspect that a pandemic involving social distancing might reinforce isolation for older adults, an adaption of our usual and customary protocols might reflect that web-based groups may afford subjects the comfort of being alone in their homes, or rooms, while at the same providing for time together with others, personal and professional carers, in the context of a screen. Where there are re-routings of this sort, however, there can be surprises in the data. There are spaces for alteration.

Finally, bringing the reality of the COVID 19 pandemic into our research thinking may call upon us as a global community of music, music therapy and neuroscience researchers to expand our domains and cohorts types, directing our protocols to address novel or under-researched topics, such as the impact of music on pulmonary function. Recent music-based protocols for children and adults with chronic respiratory diseases, for example, show promise (Canga et al., 2015; Loewy et al., 2020) despite the scarcity of clinical trials on the topic. Since breath control is reliant on a brainstem neuronal network that can be strengthened with predictability and rhythmic reinforcement, perhaps landing ourselves in projects involving music and pulmonary function might gain traction and align opportunities for innovative funding support.

### Response to Limitations

Several studies point out that researchers are at high risk of suffering anxiety and depression (Levecque et al., 2017; Evans et al., 2018; Duffy et al., 2019). The pandemic forces us to stop most research projects, and this has a subsequent negative impact on productivity. This is particularly relevant for young scientists that are pursuing their Ph.D. studies or are on a path leading toward a tenure-track position. On the other hand, quite a few researchers in the music, music therapy and neuroscience field are still going forward resiliently. Along with the rest of the world, they seem to have embraced technology and cell-phone based modes of communication, as they have transitioned feasible interventions from in-person to online modes of delivery.

Luckily, music is a mode of communication with some aspects that can be easily transferred to the virtual domain. For example, gamification of interventions and home-based interventions administered through apps (Benveniste et al., 2008; Benveniste, 2010; Boulay et al., 2015; Bégel et al., 2018; Ramirez et al., 2020) are currently being used with more acceptance and may be a viable solution for certain kinds of interventions moving forward. Nevertheless, while during the pandemic this approach has certainly involved a learning curve for many, with collaboration and some effort it has proved to be a valuable tool. This kind of adaptation or modification may not only help maintain research momentum but can provide much needed relief during this difficult period.

## The Way Forward

As research continues for some in its adapted state, and/or when it resumes for others, we will need to be mindful and sensitive. We need to have an understanding of how time and temporary cessation, as well as limited mobility and social restrictions have affected our projects. At the same time, we need to discover mechanisms for accurate analysis and best reporting of these disruptions, as well as markers that will inevitably determine when to begin research, and/or resume and adhere to the original and/or alter the interventions and data collection in our projects. Do we perhaps need to be more cautious in these times than the general health and safety guidelines dictate, especially when we deal with vulnerable populations and at-risk groups? Which research takes priority within labs when measurement times get bottlenecked? These are some critical questions to answer, and the only way to overcome these issues is through communication within the scientific community, and with our clinical partners as well. This occurs, most surely, with patience.

During the COVID-19 lockdown, the time some researchers “lose” in data collection is time that fortunately can be used for in-depth literature review catch-up, for data analysis, reading and writing. As mentioned, for some the research continues in a temporarily altered format. For many others this period, while being in many ways stressful and demanding, has also been a welcome opportunity to catch up on those secondary research tasks, such as analyzing old data and finishing up old manuscripts, for which time under usual circumstances, may be difficult to find. Overall, this exceptional period can give us a moment to become creative and adjust current projects, or to slow down and reflect, and in all cases, to consolidate and understand nuances that may have perhaps been otherwise missed.

As music researchers, we can study closely what pandemics do to people, how they have reacted, what kind of problems they have faced, what resilience they have shown, and what role music and its related technological, biological and psychological extensions might play in this situation. During the COVID-19 crisis, news images and videos from Italy, Spain and elsewhere featured people quarantined to their homes playing music and singing together from their open windows and balconies to help cope with the emotional distress and social isolation; a testament to the emotional and social power of music.

Importantly, in these times, new music intervention projects and also new target groups requiring support can be identified. One such group are the frontline workers and medical staff who face unparalleled stress and burn out from shouldering staggering responsibility. In New York city, for example, the trend for virtual “music rooms” for staff is a tradition renewed from 9 to 11, which showed the impact of music therapy on another kind of trauma (Loewy and Frisch-Hara, 2002). The treatment of trauma is a growing discipline, and is beginning to be embraced in the music neuroscience world. Pandemics are fertile ground for the continuance of such important work (Porges and Rossetti, 2018). Another important group to turn our research efforts upon are the elderly isolated in their homes, for whom online music interventions could be explored as tools to lift mood, soothe anxiety, and provide social contact.

Moving forward with hope rests on an assumption that there will always be support for improving the human condition. As researchers working in this exceptional situation, we need to be patient and open in our views and also perhaps a bit more permissive when it comes to meeting our own deadlines and achieving goals. For instance, we may have to make addendums for reporting confounding variables in the pursuit of our research objectives as we think of new ways to implement our research. We also need to understand that people react differently to crisis. We have to respect this and take it into account when working together. On the other hand, it is important to keep pressing forward, to be proactive, and make the necessary comprises when needed, rather than to bury our heads in the sand and simply wait for things to change or get better.

Finally, now more than ever, it is important to come together as a community, to interact, learn from each other, and identify new research avenues where music could be used to combat among others the emotional and social burden caused by COVID-19. An instance of this coming together is the Musicovid network (which can be accessed at https://www.aesthetics.mpg.de/forschung/abteilung-musik/musicovid-an-international-research-network.html) that compiles and makes available to the public domain research conducted in this area. This is an exemplary model of community convergence, research communication and support, showcasing an effective way forward. Taking care of ourselves and of the people we work with is and should be paramount in these difficult and extraordinary times we live in, hence a must for our research community, too.

## Data Availability Statement

All datasets presented in this study are included in the article/supplementary material.

## Author Contributions

EP and FZ drafted the main body of the manuscript. TS, JG-S, RR, MT, and JL contributed parts and commented on the manuscript. EP, FZ, and JL edited and proofread the manuscript. All authors approved the final submission.

## Conflict of Interest

The authors declare that the research was conducted in the absence of any commercial or financial relationships that could be construed as a potential conflict of interest.
